# Comparison of tonic spinal cord stimulation, high-frequency and burst stimulation in patients with complex regional pain syndrome: a double-blind, randomised placebo controlled trial

**DOI:** 10.1186/s12891-015-0650-y

**Published:** 2015-08-25

**Authors:** N. Kriek, JG Groeneweg, DL Stronks, FJPM Huygen

**Affiliations:** Center for Pain Medicine, Erasmus University Medical Center, Postbox 2040, 3000 CA Rotterdam, The Netherlands

## Abstract

**Background:**

Complex Regional Pain Syndrome (CRPS) is a disabling disease that is sometimes difficult to treat. Although spinal cord stimulation (SCS) can reduce pain in most patients with CRPS, some do not achieve the desired reduction in pain. Moreover, the pain reduction can diminish over time even after an initially successful period of SCS. Pain reduction can be regained by increasing the SCS frequency, but this has not been investigated in a prospective trial. This study compares pain reduction using five SCS frequencies (standard 40 Hz, 500 Hz, 1200 Hz, burst and placebo stimulation) in patients with CRPS to determine which of the modalities is most effective.

**Design:**

All patients with a confirmed CRPS diagnosis that have unsuccessfully tried all other therapies and are eligible for SCS, can enroll in this trial (primary implantation group). CRPS patients that already receive SCS therapy, or those previously treated with SCS but with loss of therapeutic effect over time, can also participate (re-implantation group). Once all inclusion criteria are met and written informed consent obtained, patients will undergo a baseline assessment (T0). A 2-week trial with SCS is performed and, if successful, a rechargeable internal pulse generator (IPG) is implanted. For the following 3 months the patient will have standard 40 Hz stimulation therapy before a follow-up assessment (T1) is performed. Those who have completed the T1 assessment will enroll in a 10-week crossover period in which the five SCS frequencies are tested in five periods, each frequency lasting for 2 weeks. At the end of the crossover period, the patient will choose which frequency is to be used for stimulation for an additional 3 months, until the T2 assessment.

**Discussion:**

Currently no trials are available that systematically investigate the importance of variation in frequency during SCS in patients with CRPS. Data from this trial will provide better insight as to whether SCS with a higher frequency, or with burst stimulation, results in more effective pain relief.

**Trial registration:**

Current Controlled Trials ISRCTN36655259

## Background

Complex Regional Pain Syndrome (CRPS) is defined as a collection of locally appearing painful conditions following a trauma, which mainly occurs distally and exceeds in severity and duration the expected clinical course of the original trauma; this often results in considerably restricted motor function and is characterised by a variable progression over time [1]. The diagnosis of CRPS in the acute phase is made with the aid of the new criteria of the International Association for the Study of Pain (IASP). These criteria have a high sensitivity of 0.99 and a much improved specificity of 0.79 compared with the previously used diagnostic criteria, such as the Veldman criteria and the Orlando criteria [2–4].

The natural course of the disease is not always favourable, even with a multidisciplinary approach and the use of evidence-based guideline-supported treatment algorithms. CRPS has an estimated incidence of 5.46–26.2 per 100,000 person years (95 % CI: 23.0–29.7) [5, 6] and a prevalence of 20.57 per 100,00. Women are affected more frequently than men (with a ratio of 3.4–4.0:1) and CRPS occurs more often in the upper extremities [5, 6]. The main initiating cause is a fracture, but other traumatic or spontaneous onsets are well documented [3, 5].

Although the pathophysiology of CRPS is incompletely understood, several mechanisms are proposed to contribute to this disease such as inflammation, vasomotor dysfunction, and involvement of the central nervous system with features of central sensitisation and neuroplasticity [7].

Some patients with diagnosed CRPS are unresponsive to any conventional therapy, such as physical therapy, medication and invasive interventions such as sympathetic blockade. In these cases there is a clear indication for spinal cord stimulation (SCS) as a last resort therapy to reduce pain. SCS has been performed in many CRPS patients and has shown an evident and clinically relevant pain reduction that can be maintained for a prolonged period of time [8–12].

The exact mechanism of action that occurs during SCS, which results in pain relief, has not been fully elucidated. However, it is known that for neuropathic pain in general, SCS decreases the hyperexcitability of the multimodal wide dynamic range neurons (WDR), increases the release of gamma-aminobutyric acid (GABA) in the dorsal horn and, subsequently, decreases the release of glutamate. Other mechanisms proposed to be involved are activation of an inhibitory descending pathway and involvement of the cholinergic system. Several CRPS-specific mechanisms of SCS are considered to play a pivotal role, such as inhibiting the hyperexcitable central neural circuitry, a decrease in the efferent-sympathetic output and by antidromic activation which, in turn, causes the release of several vasoactive substances, such as nitric oxide, substance P and calcitonin gene-related peptide [13–15].

However, despite these successes some CRPS patients are not responsive or, in others, the pain-reducing effect diminishes over time due to tolerance [9–12]. It is suggested that pain relief in some of these tolerant non-responsive patients can be recaptured by increasing the SCS frequency to 250 Hz and beyond [16]. Also, burst SCS is a novel stimulation modality that can reduce pain, but has not yet been investigated in patients with CRPS [17, 18]. Further research is needed to gain more insight into the (patho)physiological mechanisms of CRPS and to elucidate the effects of SCS when stimulating with different frequencies and waveforms.

### Aim

This study aims to compare the effects of five different SCS modalities in patients with CRPS and determine which frequency is most effective in terms of pain reduction; these frequencies are standard stimulation with 40 Hz , 500 Hz, 1200 Hz, burst and placebo stimulation.

## Methods

### Study design

**Fig. 1:**
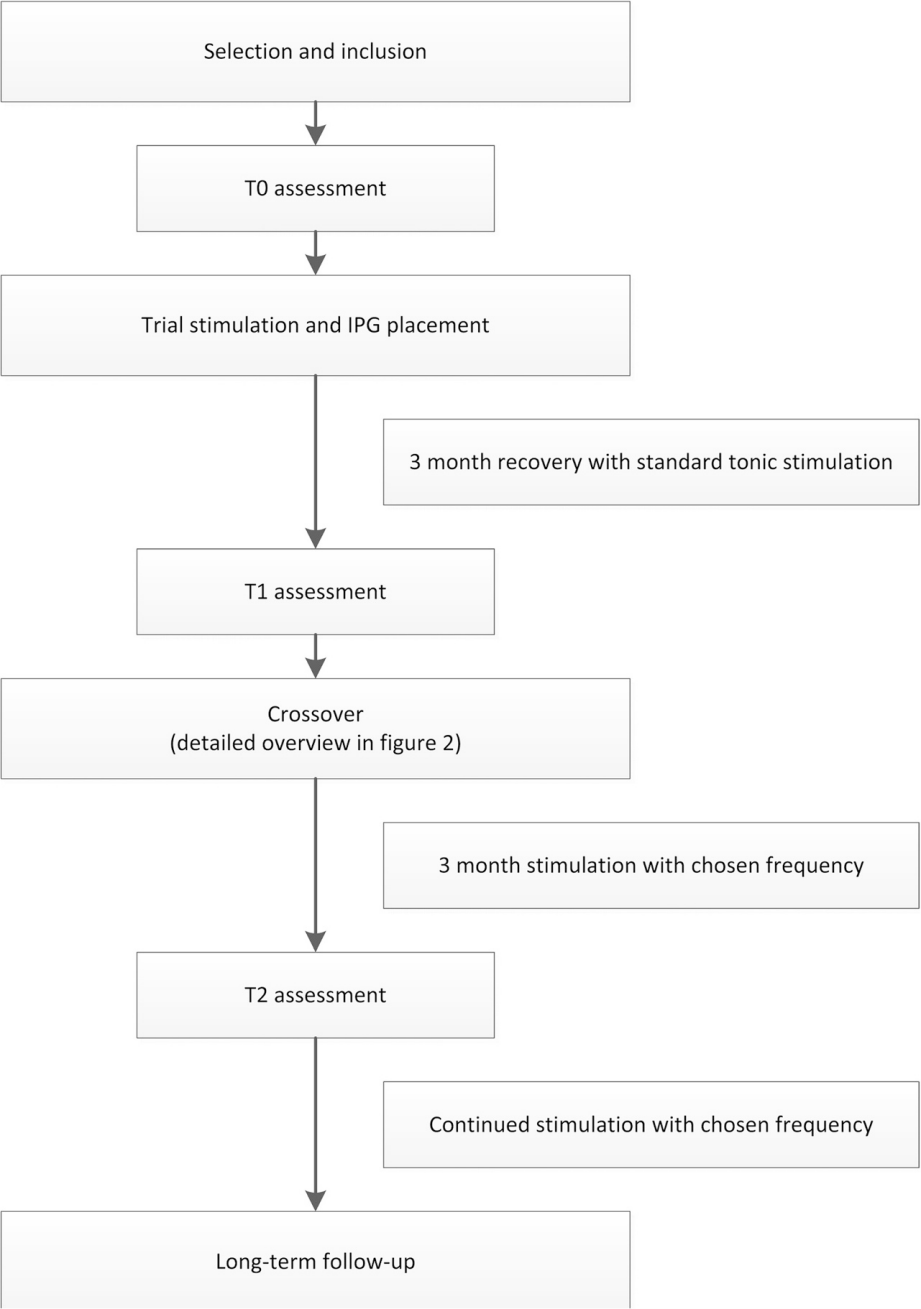
Flowchart of the entire study

**Fig. 2:**
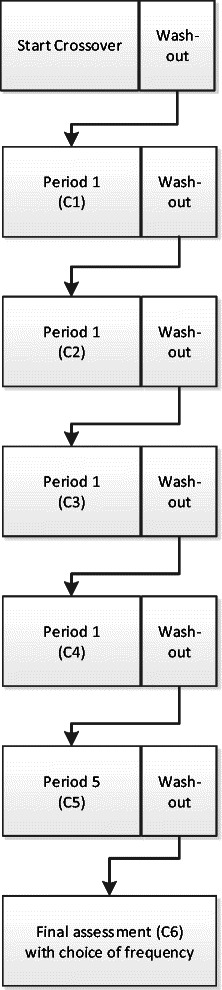
Detailed flowchart of the crossover period

This is a prospective randomised, double-blind and placebo-controlled crossover trial. The study is a multi-centre trial including one university hospital and four regional hospitals located throughout the Netherlands. Figure 1 provides an overview of the study and Fig. 2 a detailed overview of the crossover trial.

### Study population

Patients considered for enrollment in this trial must be diagnosed with CRPS that is in accordance with the endorsed IASP criteria at the time of disease onset. We defined two groups of patients that can take part in this trial. The first group (primary implantation group) consists of patients with chronic CRPS who are unresponsive to conventional therapies (e.g. pharmacologic treatment, interventional blockade of the sympathetic nervous system, or physical therapy aimed at increasing or regaining functionality by training programs). The second group (re-implantation group) consists of patients who already have SCS therapy, or were treated with SCS in the past but with loss of therapeutic effect over time. In the re-implantation group it is imperative that the paresthesia, that is or was felt under SCS therapy, has adequate overlap with the painful area.

### Inclusion and exclusion criteria

Eligibility for SCS is in accordance with the Dutch CRPS and Neuromodulation Guidelines. These guidelines state that patients must have a mean pain intensity of a least 5, as measured on a visual analogue scale (VAS; range 0–10), a CRPS duration of at least 12 months, and no lasting success or complications with conventional therapy. An additional requirement for this study is that the CRPS is located in one extremity only. Exclusion criteria are: anticoagulant drug therapy or disturbed coagulation, age < 18 years, pregnancy, ICD or pacemaker, life expectancy < 1 year, lack of cooperation of the patient, drugs, medication or alcohol addiction, and immune-compromised patients.

### Ethical approval

Approval from the Medical Ethics Committee of Erasmus Medical Center Rotterdam was obtained in August 2011 (Registration number: MEC-201 1-012). The trial was subsequently registered in the Current Controlled Trials register (ISRCTN 36655259). Written informed consent is obtained from all patients before enrollment into the trial.

### Primary outcome parameters

The main study parameters are pain reduction and patient satisfaction. The pain scores are measured with the McGill Pain Questionnaire-Dutch Language Version (MPQ-DLV) [1, 19] and a 4-day VAS diary measurement is used to monitor pain intensity during the morning, afternoon and evening. The Global Perceived Effect (GPE) is measured by two questions on a 7-point Likert scale [9]. The VAS score is considered the main primary outcome parameter out of these three. All questionnaires are completed by the patient.

### Secondary outcome parameters

Secondary outcomes consist of detecting temperature asymmetry by using video thermography imaging [20–27], and electrical quantitative sensory testing (e-QST) together with the conditioned pain modulation (CPM) [28–32]. Current Perception Threshold (CPT), Pain Perception Threshold (PPT) and Pain Tolerance Threshold (PTT) are measured with e-QST and provide more insight into the disease characteristics, such as hyperalgesia, hypoalgesia and allodynia. The CPM paradigm will provide further details on the patient’s pain suppression system.

A MicroFet Dynamometer® is used to measure muscle strength of the flexors and extensors of the elbow in case of the upper extremity, and of the knee and the foot in case of CRPS in the lower extremity. The grip strength and the pinch strength is determined by means of a Jamar Hydraulic Hand Dynamometer® and a Jamar Hydraulic Pinch Gauge®, respectively. The pinch strength is measured in two ways: thumb tip to index fingertip, and thumb pad to lateral aspect of middle phalanx of index finger; the biceps and the triceps. In addition, a walking test is performed only in patients that have lower extremity CRPS. This test records how long it takes to cover a 10 m distance when walking at a comfortable and maximum walking pace. Furthermore, the maximum walking distance is assessed during a 2-min walk where the patient is instructed to cover as much distance as possible within these 2 min [33–40].

The types of activity and physical activity level (PAL) of a patient is continuously measured with the VitaMove system (2 M Engineering, The Netherlands) for multiple days (2–3 day), providing a detailed and continuous report on the patient’s energy expenditure, motions and postures over the course of several days [41–46]. This device is capable of interpreting various positions like sitting, standing and lying down as well as discerning various activities like walking, running, cycling or general motion of the arms, legs and trunk. The PAL can be estimated with the VitaScore software that calculates the energy expenditure with the aforementioned activities and the patients characteristics like weight, height, age and sex to estimate the basal metabolism. The energy expenditure can provide information on the level of activity of a patient that ranges from extremely inactive-extremely active.

Artificial skin blisters are created to measure whether local inflammation is still present in these patients before SCS and the effects stimulation therapy might elicit on the inflammatory profile [47–56]. A blood test is performed to assess the outcome of the endogenous opioids (such as dynorphin, endorphin and encephalin) before and after SCS [57–59]. An additional consent must be signed by the patient in order to perform both the blood and artificial skin blisters test.

Multiple questionnaires are filled in by the patient throughout the course of the study to monitor the various dimensions (Table 1). These include *psychological and social evaluation:* Inventory for Social Support (ISS) [60], Pain Catastrophizing Scale Dutch Version (PCS-DV) [61–63], RAND-36 [64–66], the Hospital Anxiety and Depression Scale (HADS) [67, 68] and the Tampa Scale for Kinesiophobia (TSK) [69, 70]. *Physical activity:* the Disabilities of Arm Shoulder and Hand Questionnaire (DASH) [42, 71, 72] when the upper extremity is affected, and the Walking Ability Questionnaire when the lower extremity is affected [73–75]. *Economic evaluations:* The cost analysis is performed from a societal viewpoint in collaboration with the Institute for Medical Technology Assessment of Erasmus University Rotterdam. Three categories of costs are distinguished: 1) Direct costs within the healthcare system, 2) Direct costs outside the healthcare system, and 3) indirect costs outside the healthcare system. These data are collected using diaries and the Trimbos/iMTA questionnaire for Costs associated with Psychiatric Illness (TiC-p) and health consumption, simultaneously with the clinical data and quality of life data. The costs are valued using the Dutch guidelines for cost analysis in healthcare (Health Care Insurance Board).

**Table 1:** Data collection overview throughout the entire study

	T0	T1	C1	C2	C3	C4	C5	C6	T2
*Medical History*	x								
*Physical examination*	x	x							x
*Stimulation evaluation*		x	x	x	x	x	x		x
*Pain diary*	x	x	x	x	x	x	x		x
*McGill Pain Questionnaire*	x	x	x	x	x	x	x		x
*Global Perceived Effect*		x	x	x	x	x	x		x
*Final assessment frequency chosen*								x	
*HADS*	x	x							x
*RAND-36 / SF-36*	x	x							x
*PCS*	x	x							x
*ISS*	x	x							x
*TSK*	x	x							x
*DASH*	x	x							x
*Walking Ability Questionnaire*	x	x							x
*Prodisq A-E*	x	x							x
*TiC-p and Healthcare consumption*	x	x							x
*Thermographic imaging*	x	x							x
*Muscle strength*	x	x							x
*Walking Test (if applicable)*	x	x							x
*QST*	x	x							x
*CPM*	x	x							x
*Blood and Blister test (optional)*	x	x							x
*Physical activity monitoring*	x	x							x

Productivity costs: The productivity costs are defined as the costs of absence from paid work due to CRPS, including the impact of compensation mechanisms, the cost of efficiency losses (productivity costs while working at a slower pace) and the costs of hindrance at unpaid work. For this, the three modules of the PRODISQ questionnaire for measuring and valuing productivity costs for unpaid work are used [76, 77]. For costs related to unpaid work we use a module of the Health and Labour Questionnaire (HLQ) [78]. For the valuation of productivity losses, the friction cost method is used, taking into account that in a production process everyone is replaceable after a certain period (the so-called friction period). For the valuation of each hour of productivity lost (and the length of the friction period) we use the guidelines as presented in the costing manual [79].

Medication consumption is assessed by taking an inventory of all (pain)medication and dosing the patients use at the time of the T0, T1 and T1 assessment. The pain medication will be stratified into the various medication groups and a the sum dosage of the various pain medication groups will be calculated.

### Sample size calculation

By means of MANOVA for repeated measurements within factors, the potential differences in effect of the five different stimulation modalities will be assessed. A minimal detectable effect size (f) of 0.15 on the main primary outcome parameter pain intensity (VAS) was chosen. The required minimum number of participating patients is negatively associated with the correlation between measurements during the cross-over. Unfortunately, no data of previous research on this correlation were available at the time of setting up this study. Therefore, we made an conservative guess and a correlation of 0.6 was chosen. Thus, assuming a power of 0.8 and a significance level (σ) of 0.05 , an a priori sample size of 48 is required.

### Randomisation and blinding

Randomization was performed with a computer based program at the beginning of the trial and no blocks were used since this is not viable in our study design. In our study design all patients in the crossover will be subjected to all five stimulation modalities in random order, resulting in 5! (120) different orders in which the stimulation modalities can be programmed. Only 48 (the required sample size) of these 120 potential different orders are selected at random by the computer to be used.

The specific order of the programming for an individual patient is revealed to the local SCS programmer only during each of the five crossover periods. The patient is blinded for the intervention by means of a mask that cannot be seen through. The outcome assessments at the end of each 2-week period are done by means of questionnaires. The statistician who performs the analysis of the measurements and questionnaires is blinded for the allocation key for the treatments for each patient.

### Statistical analysis

Data are analyzed using the latest version of IBM SPSS Statistics. Descriptive statistics are used to determine the frequencies of the demographic and secondary outcome parameters, and to describe measures of central tendency and of dispersion, dependent on the shape of their distribution. The Kolmogorov-Smirnov test is used to analyze whether or not the scores on the these parameters are normally distributed. The outcome parameters are analyzed using Mixed Linear Models for repeated measurements within factors model. These Mixed Linear Models model require normality of residuals. These models are however relatively robust against violations of this assumption [80]. Therefore, the results are presented as mean ± the standard deviation (SD). Furthermore, a Bonferroni test will be performed in the pairwise comparison of the modes of stimulation only if the result of the overall analysis results in the rejection of the H0-hypothesis of no significant differences between the modes of stimulation. Therefore, to test this H0-hypothesis we will use the traditional rejection zone of 5 %. The primary analysis will not make a distinction between primary implantation group vs. re-implantation group since the vast majority of the included patients will be SCS naïve. This matter should than be dealt with in a post hoc testing.

### Pre-trial assessment

Patients with an indication for neuromodulation are referred by their physicians to the SCS nurses for further evaluation, and to provide them with additional information about the procedure and lifestyle limitations. Also, a psychologist will screen the patient to rule out any psychological contraindication that might influence the outcome of the SCS trial. The patients are contacted by the researcher for trial information and enrollment once all the clinical inclusion criteria for SCS treatment have been met. After obtaining written obtained consent from the patient, the next phase in the trial can commence. It is noteworthy that patients who decline to participate in the trial will still be treated with regular SCS therapy.

### Baseline measurement T0

The patient is invited to our medical centre to participate in various test that are conducted as well as medical history and physical examination. All questionnaires must be filled in by the patient prior to this visit. Table 1 presents an overview of the tests and measurements that are performed during the entire course of this trial. The results of the T0 tests will serve as a baseline and all subsequent evaluations will be compared with these results. An additional requirement for the patients that still have an active SCS device is to switch off the stimulation for at least 1 week before T0. This will provide objective insight into the disease characteristics and pain that would otherwise be masked by the SCS therapy. Figure 1 presents a complete overview of the various phases in this trial.

### Trial stimulation and implantation

The trial stimulation is conducted at the various hospitals attended by the patients and lasts for 2 weeks. Only experienced physicians are allowed to perform implantation. During the test stimulation the patient is positioned in the prone position and spinal column landmarks are identified with fluoroscopy. Local anaesthetics are used during this procedure; however, a mild sedative or short-acting opioid can be used without compromising the patient’s ability to cooperate during the intra-operative testing. The surgical procedure used is left to the discretion of the physician in charge.

The cylindrical percutaneous Octrode™ lead (St. Jude Medical, Plano, TX, USA ) is primarily used in all patients to gain unilateral stimulation and is positioned under fluoroscopic guidance. The correct position is determined by means of intra-operative stimulation and is deemed successful if the induced paresthesia has an adequate overlap with the painful area. The lead is sutured to the fascia using an anchor. An extension cable is attached to the lead and is tunneled under the skin towards a suitable exit site. A temporary battery is attached to the external part of this extension cable during the trial period.

The parameters used for stimulation is up to the SCS nurse in charge, except for the mandatory frequency setting of 40 Hz. All other parameters (such as electrode configuration, pulse width and current output levels) are the result of the individual clinical paresthesia mapping. The trial period will generally last for 1 or 2 weeks and is considered successful if a patient has >50 % pain reduction on a VAS and/or has stated that the symptoms are much improved. All patients with a successful trial receive a permanent rechargeable internal pulse generator (IPG) from the Eon family line (St. Jude Medical, Plano, TX, USA) under general anaesthesia. The trial extension cable is removed and replaced with a new extension cable that is connected to the IPG. In some instances, a direct connection with the lead and IPG can be made if the length of the lead is sufficient. The pocket location for the IPG is free of choice and can either be in the lower abdomen or in the gluteal are. All materials are removed if a trial is unsuccessful and the patient is then excluded from further follow-up during this trial. The standard stimulation setting is further optimised during the regular checkups in the subsequent weeks. Antibiotic prophylaxis is given during both the trial and implantation in accordance with the local hospital antimicrobial guidelines.

### Follow-up measurement T1

Three months after the IPG implantation and therapy with normal tonic 40 Hz SCS, another assessment (T1) takes place. This serves as an evaluation of the effects of regular SCS on the previously described tests (Table 1). Enrollment into the crossover part of this trial can only commence after completion of this phase.

### Crossover

The crossover period consists of five periods (each lasting for 2 weeks) in which the 5 different frequencies are tested. After each period, a small assessment (S1-S5) is done to evaluate the stimulation effects on pain and GPE. A 2-day wash-out period, during which the stimulation is switched off, is incorporated at the beginning of the first 2-week period (S1) and at the end of each period. The purpose of a wash-out period is to objectively determine the baseline pain levels and symptoms of CRPS when they are not masked by SCS, and to minimise the carryover effects of the previous period into the next period. The small assessments (S1-S5) comprise a stimulation evaluation, a pain diary, the McGill Pain Questionnaire and the Global Perceived Effect. Stimulation evaluation requires a patient to keep a stimulation diary in which they must answers four questions; (1) the effect(s) of stimulation on pain in the CRPS effected area, (2) if they feel paresthesia and if so where do they feel it and how is the stimulation described, (3) how they would rate the ease of use of the stimulation, (4) the advantages and (5) the disadvantages per stimulation modality. A detailed overview of the crossover period is provided in Fig. 2.

During the final evaluation (S6) a decision is made by the patient regarding which stimulation modality is to be used for the subsequent 3 months at the end of these 5 periods. This decision is mainly based on pain reduction, but also on other CRPS-related symptoms and practical matters related to the stimulation. The patient continues with their stimulation of choice for another 3 months. A switch of stimulation within these 3 months is possible in certain cases.

### Programming the various frequencies

The five stimulation modalities are all programmed in such a way the maximum effect pain be obtained. Standard 40 Hz stimulation will be performed above threshold which generates paresthesia in the affected area and is in accordance with normal practice. Both 500 Hz and 1200 Hz will be above threshold stimulation and will also generate paresthesia. At the time this study protocol was postulated there were no other reports available on how best to program 500 and 1200 Hz SCS based on mechanisms of action. Thus, in absence of evidence stating the contrary, we decided to program 500 and 1200 Hz SCS like standard 40 Hz stimulation with the intention to induce paresthesia and thus being an above threshold stimulation. Placebo stimulation is performed with the IPG switched off and thus does not generate paresthesia. The rationale is to evaluate how big a part of the pain relief could be attributed to the placebo effect. Burst stimulation is performed with fixed stimulation parameters and the intensity of the stimulation will be sub-threshold and therefore is does not generate paresthesia. The fixed parameters of burst stimulation are: an overall frequency of 40 Hz per burst complex. Each burst complex delivers 5 spikes with a frequency of 500 Hz and a pulse width per spike of 1 ms and an inter-spike interval of 1 ms. The charge is balanced during the 15 ms pause between the burst complexes.

### Follow-up measurement T2

The T2 assessment is performed when a patient has completed the 3-month period with the frequency of choice. This T2 evaluation enables us to compare all the primary and secondary outcomes parameters with the T1 assessment with standard SCS and the baseline T0 assessment prior to SCS therapy. The patient is actively asked whether they want to continue with the SCS program they have chosen, or want to switch to another one based on the crossover results.

### Trial completion

After completion of this trial the patient will continue with the chosen stimulation and a clinical follow-up is performed once a year by the physicians and nurses.

## Discussion

Data obtained from this trial will provide better insight as to whether SCS, as it is currently applied, is the best available therapy modality, or whether stimulation with a higher frequency or with burst stimulation results in better pain relief. Another result might be that these new modalities of stimulation are equally preferred by the patients. Irrespective of the outcomes, this trial will provide new insights into the mechanisms of action involved in SCS treatment and, particularly, in patients with CRPS.

For this trial, standard SCS was defined as stimulation with 40 Hz and corresponds with the normal stimulation setting used in our daily practice. The choice to select 500 Hz was based on studies reporting that blood flow and endogenous opioid release are at their maximum while applying SCS at this frequency [57, 59, 81]. The 1200 Hz stimulation was chosen because it is the maximum that can be generated with this IPG. Burst stimulation was selected because the stimulation differs significantly from tonic stimulation in terms of waveform and working mechanism. It is reported that burst SCS can effectively reduce pain in patients with neuropathic pain [17, 18, 82–84].

High-frequency SCS (HF-SCS) with 10,000 Hz is also available and multiple studies have shown its effect in pain patients and its ability to reduce pain over a longer period of time [85, 86]. Our 1.2 kHz stimulation does not approach the 10 kHz used in the latter studies, but no clinical studies are available that compare these frequencies. However, some animal studies have compared the effects of normal frequency SCS with different HF-SCS settings. Song et. al. compared conventional suprathreshold stimulation of 50 Hz with that of subthreshold HF-SCS at 500 Hz, 1 kHz and 10 kHz and concluded that the hypersensitivity after nerve lesions was reduced in all the HF-SCS groups and that the reduction was equal to that of the normal SCS group [87].

Burst stimulation is a new concept which requires a totally different programming algorithm in daily practice. The stimulation must be programmed to a sub-threshold current output level so that a patient will not experience paresthesia. This paresthesia free ‘side-effect’ can be used to incorporate a placebo element into the trial where paresthesia cannot be felt because the stimulation is switched off.

The question remains if a 2 week test period per frequency along with a 2 day wash-out to counteract the possible carryover effects of the various frequencies are enough. In clinical practice a patient can conclude whether the SCS is beneficial to him/her within a two week period during trial SCS. We choose to maintain the 2 week per period analogy in the crossover period which should provide the patient with enough time to decide if one of the test frequencies is beneficial or not. Moreover, by increasing the crossover period to 3 weeks or even longer the possibility of patient withdrawal from the study increases in case one of the test stimulations does not provide (sufficient) pain relief. This is further supported by what clinicians hear from patients that the pain returns when the stimulation is switched off. Some experience an instantaneous increase in pain while others report that it takes a few minutes or a few hours before the pain has fully returned, but never longer than 24 h. On these arguments we decided on a wash-out period of 2 days between the various stimulation modalities to be tested [88–90].

A potential pitfall in the design of the present trial that might influence the patient’s choice, is that the patients are preconditioned with standard stimulation for the first 3 months. On the other hand, if they do choose a SCS setting other than the standard one, it is a choice they make despite the odds being in favour of the standard stimulation.

Bias and in particular attrition bias is a serious problem that could affect the results of the trial. In our trial attrition bias will be handled by describing all patients that have dropped out of the trial before its completion and the reasons for dropping out/trial exit will be documented and provided in a flowchart in a separate manuscript with the results of this trial. The flowchart will adhere to the requirements of the CONSORT statement for transparent reporting of trials. Furthermore, data will be analysed using linear mixed models. Hence we can use all of the data, i.e., if a score is missing, it has no effect on other scores from that same patient.

With the results obtained from this study, that focuses solely on patients with CRPS, we aim to increase knowledge on the effects of SCS in this patient group. However, caution is advised when extrapolating these results to other neuropathic pain states because of the single-disease inclusion approach.

Patient recruitment and inclusion started in August 2011 and the trial is currently ongoing. Completion of the first part of the study (T0, T1 and crossover) is expected in early 2015. The completion of T2 and long-term data collection is expected towards the end of 2015.

## Abbreviations

SCS: Spinal cord stimulation
CRPS: Complex regional pain syndrome
RCT: Randomized Controlled trial
IASP: International association for the study of pain
IPG: Internal pulse generator
Hz: Hertz
MPQ: McGill Pain Questionnaire
GPE: Global Perceived Effect
HADS: Hospital anxiety and depression scale
PCS-DV: Pain catastrophizing scale Dutch version
ISS: Inventory for social support
TSK: Tampa scale for kinesiophobia
DASH: Disabilities of arm shoulder and hand questionnaire
TiC-P: Trimbos/iMTA questionnaire for costs associated with psychiatric Illness
PRODISQ: Productivity and disease questionnaire
e-QST: Electrical quantitative sensory testing
CPM: Conditioned pain modulation
CPT: Current perception threshold
PPT: Pain perception threshold
PTT: Pain tolerance threshold
